# Patient Characteristics in Oral Cancer Staging

**DOI:** 10.3389/froh.2022.923032

**Published:** 2022-06-09

**Authors:** Matthew J. Hoffman, Demetria D. Hale, Elijah W. Hale

**Affiliations:** ^1^University of North Carolina Greensboro, Greensboro, NC, United States; ^2^High Point University, High Point, NC, United States; ^3^School of Medicine, University of Colorado, Aurora, CO, United States

**Keywords:** oral cancer, epidemiology, risk factors, screening, dental health

## Abstract

**Background:**

Oral cancer is a largely preventable malignancy with many modifiable risk factors, such as tobacco use and proper oral hygiene. Early detection of oral cancer is an important goal for oral healthcare providers, as survival rates for oral cancers diagnosed at an advanced stage are less than half the rates for cancers diagnosed in early stages. As many patients are asymptomatic in early stages, it is crucial for oral healthcare providers to have a high index of suspicion while treating patients at risk for late diagnosis.

**Objectives:**

To identify characteristics associated with early vs. late stage diagnosis of oral cancer.

**Methods:**

We performed a retrospective chart review using the TriNetX database. We identified two cohorts of interest: patients with an initial diagnosis of stage 1 oral cancer, and patients with an initial diagnosis of stage 3 or 4 oral cancer. Statistical comparison of cohort characteristics was completed through the TriNetX statistical software platform.

**Results:**

We identified 386 patients diagnosed at stage 1 and 869 patients diagnosed at stage 3 or 4. We identified several characteristics not previously reported in the literature. Race, BMI between 20 and 29, malnurition, anemia were all associated with late stage diagnosis. Certain medications were also associated with late stage diagnosis, such as heparin derivatives and diclofenac. Our findings also reinforced prior research for characteristics such as nicotine use and ethnicity.

**Conclusion:**

Our findings offer new characteristics that may aid oral healthcare providers in detecting oral cancer at an early stage. Increasing provider awareness of factors that they may not have considered previously could increase the rates of early stage cancer detection, improving overall patient mortality and curative outcomes.

## Introduction

Oral cancer is a largely preventable malignancy with many modifiable risk factors, such as tobacco use and alcohol use [1]. Early detection of oral cancer is an important goal for oral healthcare providers, as survival rates for oral cancers diagnosed at an advanced stage are less than half the rates for cancers diagnosed in early stages [1]. Past epidemiological studies into oral cancer describe a growing prevalence rate, with as many as 50% of patients being diagnosed at stage 3 or 4, at which point 5-year survival can be as low as 28% 0.2 Some factors in late-stage diagnosis are unavoidable or intractable, such as provider availability or lab delay [2]. However, as many patients are asymptomatic in early stages, it is crucial for oral healthcare providers to have a high index of suspicion while treating patients at risk for late diagnosis [3].

As certain patient characteristics such as tobacco use or extensive tooth decay often lead clinicians to perform a screening examination for oral cancer, [3] it is possible that providing oral healthcare providers with information on other, less obvious risk factors may improve their clinical judgement on who to screen for oral cancer. In the past, information on less known risk factors such as human papillomavirus (HPV) infection has been successfully disseminated to the oral health community, leading to an increase in clinician identification of patients that may have progressed to an advanced stage without this education [4]. If new risk factors could be identified and shared in a similar manner, it could greatly benefit many patients. In this study, we attempt to identify novel characteristics associated with oral cancer stage at diagnosis using a large health database.

### Methods

We performed a retrospective chart review using the TriNetX database which contains over 85 million distinct patient records from 58 healthcare organization located in the United States and internationally [5]. Using the 10^th^ version of the International Classification of Diseases (ICD-10) codes, we identified two cohorts of interest: patients with an initial diagnosis of stage 1 oral cancer, and patients with an initial diagnosis of stage 3 or 4 oral cancer (Table 1). We limited the time period from January 1, 2010 to December 31, 2021. Inclusion criteria were ICD-10 codes C06 OR C14, which pertain to unspecified and ill-defined malignant neoplasms of the oral cavity, respectively. These patients were split into two groups based on the oncology detailed report, either into stage 1 or Stage 3 and 4. There were no exclusion criteria. Summary statistics were obtained for each cohort through frequency analysis. T-tests were used to compare differences between patient characteristics. Relative Risk Ratios were calculated from the given incidence of each characteristic. Statistical comparison of cohort characteristics was completed through the TriNetX statistical software platform. Significance for this study was set at *p* < 0.05. The Colorado Multiple Institutional Review Board designated this study as exempt due to the deidentified, aggregate form of data used.

**Table 1:** Early and late diagnosis of oral cancer by patient characteristics.

**Demographics**	**Early Diagnosis (Total *N* = 386)**	**Late Diagnosis (Total *N* = 869)**	**Relative risk**	***P*-value (sig: *P* ≤0.05)**
Average age	69.5 years	70.5 years	—	0.1819
Female	253 (65.5%)	611 (70.3%)	1.07	0.0924
White	224 (58%)	527 (60.6%)	1.04	0.3835
Black or African Americans	22 (5.7%)	86 (9.9%)	1.74	**0.0144**
Hispanic/Latino	11 (2.8%)	47 (5.4%)	1.93	**0.0463**
Not Hispanic/Latino	193 (50%)	424 (48.8%)	0.98	0.6927
**Diagnoses**	**Early Diagnosis (Total** ***N*** **=** **386)**	**Late Diagnosis (Total** ***N*** **=** **869)**	**Relative risk**	***P*****-value (sig:** ***P*** **≤0.05)**
BMI 30–39	21 (5.4%)	38 (4.4%)	0.81	0.4096
BMI 20–29	36 (9.3%)	117 (13.5%)	1.45	**0.0387**
Anemia	59 (15.3%)	189 (21.7%)	1.42	**0.008**
Nicotine dependence	66 (17.1%)	194 (22.3%)	1.3	**0.035**
Malnutrition	40 (10.4%)	200 (23%)	2.21	**0.0001**
**Medication types**	**Early Diagnosis (Total** ***N*** **=** **386)**	**Late Diagnosis (Total** ***N*** **=** **869)**	**Relative risk**	***P*****-value (sig:** ***P*** **≤0.05)**
Antirheumatics (Diclofenac/derivatives)	218 (56.5%)	419 (48.2%)	0.85	**0.0069**
Antipsychotics or (non-phenothiazines)	38 (9.8%)	147 (16.9%)	1.72	**0.0011**
Anticoagulants (Heparin/derivatives)	119 (30.8%)	327 (37.6%)	1.22	**0.0202**

*Bold value indicates a p value < 0.05*.

## Result

We identified 386 patients diagnosed at stage 1 and 869 patients diagnosed at stage 3 or 4. Patient demographics such as Black race and Hispanic/Latino ethnicity were found to be associated with late stage diagnosis, while age, sex, and non-Hispanic/Latino ethnicity had no significant impact on staging. Patient diagnoses were also investigated: obesity had no impact on stage at diagnosis, but a BMI within 20–29, anemia, and nicotine dependence were all associated with stage 3 or 4 oral cancer. Finally, we investigated medications. Patients with a later stage of diagnosis were more likely to use antipsychotic medications and anticoagulants, while antirheumatic medications were associated with an earlier stage of diagnosis (Table 1).

## Discussion

Our findings identified several characteristics not previously reported in the literature. Certain findings such as anemia offer a unique opportunity for provider awareness, as they are likely documented in the patient's chart and could prompt a provider to increase their index of suspicion for oral cancer. It is also notable that malnutrition was also strongly linked with late-stage diagnosis; it may be beneficial to have a higher awareness of oral cancer overall in any patient showing early signs of malnutrition, given the most common cause of anemia is nutritional deficiency [6]. Similarly, the medications identified offer a similar opportunity, as heparin was associated with a 22% higher risk of late-stage diagnosis, and a detailed medication list including anticoagulants is an important facet of proper oral healthcare.

Some of our results were unsurprising, such as the association between nicotine dependence and late-stage diagnosis. However, other findings indicate a necessity for increased screening within certain patient populations. While the connection between Black race or non-obese BMI and late-stage diagnosis may seem confusing at first, it can be explained with previous research on oral cancer suspicion within healthcare providers; within Southeast Asian countries, such as India and Indonesia, oral cancer accounts for over 25% of all new cancer diagnoses each year [7]. Similarly, diets high in sugar and processed carbohydrates are often reported to be linked to negative oral health outcomes, including oral cancer [8]. It is possible that providers of patients with obesity are more attentive to any abnormalities due to this increased baseline risk, whereas patients that seem to maintain a healthy diet are met with less suspicion for cancer by their providers.

Our study is not without limitations. The findings consisted of patient data from many different sites, including some international clinics, which may serve patients with a different standard of care than dental clinics within the United States. Secondly, due to the deidentified, aggregated nature of the data, we are unable to identify which patients were diagnosed by which providers. It is possible that other healthcare providers such as physicians contributed to the early or late diagnoses, which could explain some unexpected results like antirheumatic medications being associated with early diagnosis, as patients receiving antirheumatic medications likely interact with a physician on a regular basis.

Our findings offer new characteristics that may aid oral healthcare providers in detecting oral cancer at an early stage. As patients may remain asymptomatic even into stage 3, it is important for oral healthcare providers to be especially vigilant. Increasing provider awareness of factors that they may not have considered previously could increase the rates of early-stage cancer detection, improving overall patient mortality and curative outcomes.

## Data Availability Statement

The original contributions presented in the study are included in the article/supplementary material, further inquiries can be directed to the corresponding author.

## Ethics Statement

Ethical review and approval was not required for the study on human participants in accordance with the local legislation and institutional requirements. Written informed consent from the patients was not required to participate in this study in accordance with the national legislation and the institutional requirements.

## Author Contributions

MH, DH, and EH agreed to be held responsible for the content. EH has full access to the data within and welcomes any queries regarding the data or manuscript. MH and DH provided substantial data analysis and contributions to this manuscript. All authors contributed to the article and approved the submitted version.

## Conflict of Interest

The authors declare that the research was conducted in the absence of any commercial or financial relationships that could be construed as a potential conflict of interest.

## Publisher's Note

All claims expressed in this article are solely those of the authors and do not necessarily represent those of their affiliated organizations, or those of the publisher, the editors and the reviewers. Any product that may be evaluated in this article, or claim that may be made by its manufacturer, is not guaranteed or endorsed by the publisher.
